# The impact of sociodemographic background on clinical presentation of high-grade gliomas: a multi-institutional retrospective analysis

**DOI:** 10.1007/s11060-025-05012-1

**Published:** 2025-03-25

**Authors:** Sayak R. Ghosh, Anne R. Lally, Isabella L. Pecorari, Joshua Reynolds, Alexander Ledet, Sabrina Begley, Elizabeth Juarez Diaz, Eric Zhu, Karan Joseph, Kyle McGeehan MPhil, Michael Schulder, Tanner Johanns, Yonah C. Ziemba, Vijay Agarwal

**Affiliations:** 1 Department of Neurological Surgery, Albert Einstein College of Medicine/Montefiore Medical Center, 3316 Rochambeau Ave, Bronx, NY 10467 USA; 2 Department of Neurosurgery, Donald and Barbara Zucker School of Medicine at Hofstra Northwell, 500 Hofstra Blvd, Hempstead, NY 11549 USA; 3 Department of Oncology, Washington University at St. Louis, 660 S Euclid Ave, St. Louis, MO 63110 USA; 4 Department of Pathology and Laboratory Medicine, Donald and Barbara Zucker School of Medicine at Hofstra Northwell, 500 Hofstra Blvd, Hempstead, NY 11549 USA; 5 Montefiore Medical Center, The University Hospital for the Albert Einstein College of Medicine, 3316 Rochambeau Avenue, Bronx, NY 10467 USA

**Keywords:** Ethnicity, Glioma, High-grade, Intracranial, Malignancy, Race

## Abstract

**Purpose:**

High-grade gliomas (HGG; WHO III/IV) are among the most devastating intracranial malignancies, and outcomes may be associated with demographic, biological and environmental factors. Although research exists on the association of sociodemographic background with outcomes, the literature lacks data on the effect of sociodemographic background on clinical presentation. In this study, we aimed to examine race- and ethnicity-related differences in HGG presentation and diagnosis.

**Methods:**

We conducted a chart review of patients treated for HGG between 2015 and 2021 at three high-volume academic medical centers. A total of 314 patients were analyzed. 173 White patients were included along with 144 non-White patients, comprising of Asian (16%), Black (26%), Hispanic (9%), and other/declined (50%) race. Statistical analysis was carried out using GraphPad Prism.

**Results:**

On multivariate analysis, White race was significantly associated with a later age at diagnosis independent of IDH1 status. White patients were more likely to present with a cognitive deficit (42.3% vs. 21.1%; *p* = 0.02*), while less likely to present with midline shift (32.5% vs. 49.3%; *p* = 0.004**) and mass effect on imaging (59.8% vs. 76.1%; *p* = 0.003***). Additionally, Black patients were more likely to present with syncope (15.8% vs. 2.3% [*n* = 107]; *p* = 0.04*) and Hispanic patients were more likely to present with seizure (35.7% vs. 15.9% [*n* = 110]; *p* = 0.03*).

**Conclusion:**

White race appears to be independently associated with a later age at diagnosis of HGG. Furthermore, Black and Hispanic patients are more likely to present with severe, life-threatening symptoms. Large-scale studies are needed to elucidate race-based differences in HGG presentation to effectively predict outcomes.

## Introduction

Gliomas represent 80.7% of all primary brain malignancies [1–3]. They are typically diagnosed between the ages of 40 and 70, with a higher prevalence in males [4–6]. Gliomas are classified according to their cell of origin, and include astrocytic tumors, oligodendrogliomas, ependymomas, and mixed gliomas [1–3, 7]. High-grade gliomas (HGG) are classified by the World Health Organization (WHO) as grade III or IV based on histopathology and molecular characteristics [5, 8]. HGGs are classified by isocitrate dehydrogenase (IDH) genotype, which is associated with their cell of origin, progression, and prognosis. IDH-mutant astrocytomas tend to arise from neural precursor cells and have a better prognosis, often harboring ATRX and TP53 mutations [9]. In contrast, IDH-wild-type glioblastomas originate from differentiated glial cells, progress rapidly, and frequently exhibit EGFR amplification and TERT promoter mutations [9].

HGGs are rare, with an annual incidence of fewer than 15 cases per 100,000 people [10, 11]. Despite available treatments, high-grade gliomas (HGG) remain highly challenging to manage. Median survival is approximately 24–72 months for grade III tumors and 12–14 months for grade IV tumors, with a 5-year survival rate of only about 5% [12, 13].

The clinical presentation of gliomas varies widely and is influenced by tumor location, tumor size, rate of progression, and timing of presentation [14]. Symptoms can be broadly categorized into generalized and focal manifestations. Generalized symptoms result from increased intracranial pressure or diffuse brain involvement and commonly include headaches, nausea, vomiting, seizures, altered consciousness, neurocognitive dysfunction, and personality changes [14]. In contrast, focal deficits arise from tumor invasion or compression of specific neural structures and may present as focal-onset seizures, motor weakness, sensory deficits, aphasia, and visual spatial dysfunction [14]. Headache is the most frequently reported symptom, occurring in approximately 50–60% of patients [15]. While focal neurological deficits are more common in HGG due to their aggressive growth and mass effect, seizures are more commonly associated with lower grade gliomas [16]. Larger tumors may also induce symptoms secondary to peritumoral edema, mass effect, and elevated intracranial pressure, further contributing to neurological deterioration [16].

In the USA, incidence of glioma varies by ancestry and is greatest among European Americans [13]. The lifetime risk of developing a malignant brain tumor is twice as high in European-Americans compared to African-Americans and 25% higher than in Hispanic-Americans [17]. Glioma incidence also varies globally, with greatest rates seen in high-income countries like the USA, Canada, Australia, and Northern Europe [17]. While it is possible that biological and lifestyle differences exist between these populations and the outgroup, other reasons for this discrepancy include underreporting of cases, reduced diagnostic capability, and diminished access to healthcare in low-to-middle income countries [18, 19].

Many studies have reported differences in HGG outcomes and survival as they relate to ancestry and sex [20–23]. A prior study published using data from our institution highlighted how recurrence of HGG is delayed in Hispanic-American patients despite increased social vulnerability [24]. However, to our knowledge, this is the first multi-center study to explore the role of sociodemographic background in the clinical presentation of HGG. We found that White patients were diagnosed at an older age and were more likely to present with cognitive deficits, whereas Black and Hispanic patients were more likely to present with severe, life-threatening symptoms such as syncope and seizures, respectively. Additionally, White patients were less likely to present with midline shift and mass effects on imaging. These findings highlight the need for further research to explore the underlying factors contributing to these differences and their potential impact on diagnosis and treatment outcomes.

## Methods

### Retrospective chart review

This study was approved by the Institutional Review Board (IRB number: 2021–13288) and conducted in accordance with the ethical principles outlined in the Declaration of Helsinki. Written informed consent was not obtained from the participants given the retrospective nature of this study.

This retrospective study was conducted using data from candidate HGG patients treated at three tertiary academic centers in the United States between 2015 and 2021. Data from a total of 319 patients were collected. For the analyses in this study, patients were required to meet a set of inclusion criteria: being at least 18 years of age at diagnosis, reporting an ethnic and racial identity, and having a pathology-confirmed diagnosis of HGG (WHO III or IV).

Removal following inclusion criteria lead to a total of 317 patients for this study. Depending on data available, between 72 and 311 patients were included in each individual analysis from the total of 317 patients. Data on both race and ethnicity were drawn from the chart, with “White” and “Black” being racial identifications and “Hispanic” being an ethnic identification. IDH1 status was determined via immunohistochemical staining.

### SVI

To verify socioeconomic disadvantage in the study sample, CDC/ATSDR Social Vulnerability Index (SVI) scores were collected for each patient based upon home address. These scores draw a series of metrics from 2020 census data for the local county in which the home address is located. The overall SVI score is a composite of these metrics. The individual metrics, or sub-scores, include socioeconomic status, racial and ethnic minority status in the local population, members-of-household characteristics, transportation accessibility, and housing type. The scores for each sub-score and the overall score are reported as a percentile relative to all counties in the United States. Higher percentiles represent more severe disadvantage.

### Clinical parameters

Various clinical signs and symptoms were derived from patient notes in the chart, categorized as either being present or being absent. These symptoms included seizures, memory loss, visual disturbance, auditory disturbance, language deficit, cognitive deficit, altered mental status, motor weakness, syncope, and personality change. In addition, patient race and ethnicity was examined in comparison to the Karnofsky Performance Status (KPS) score, a clinical scoring system that assesses a patient’s functional status.

### Statistical analyses

Non-parametric Mann-Whitney U tests were used to perform univariate comparisons between groups, as multiple variables were non-normally distributed. The Fisher exact test was used to perform comparisons between categoric variables and ethnic groups. Multivariate analysis was done to examine the relationship between multiple variables and an associated outcome. GraphPad Prism and Microsoft Excel was used to analyze the data and generate figures.

## Results

### Patient cohort demographics

317 patients analyzed met inclusion criteria (adult, provided race, confirmed grade III or IV pathological diagnosis of HGG). 173 patients identified as White race, 23 patients identified as Asian race, 37 patients identified as Black race, 13 patients identified as Hispanic race, and 72 patients reported “Other” race. Notably, 29 patients who identified with “Other” race reported a Hispanic ethnicity, possibly due to the fact that their primary identification is an ethnic one rather than a racial one. Within the White group, 73 patients were female, while within the non-White group, 65 patients were female (Table 1).

**Table 1:** Patient cohort demographics; p-value of < 0.05 denotes statistical significance

		Whole Cohort		White		Non-White		*p*-value
		Mean	Std dev	Mean	Std Dev	Mean	Std Dev	
**Sample size**		317		173		144		
**Sex (% Female)**		43.2% (137)		41.6% (72)		45.1% (65)		0.5698
**Age (years)**		62.5	14.9	64.7	12.6	59.9	16.7	**0.0302**
**IDH1 Status**								
	IDH1 Wild-Type	87.1% (275)		90.8% (157)		81.9% (118)		**0.0297**
	IDH1 Mutant	10.1% (32)		6.4% (11)		14.6% (21)		**0.0234**
	IDH1 N/A	2.8% (10)		2.9% (5)		3.5% (5)		> 0.9999

**Fig. 1:**
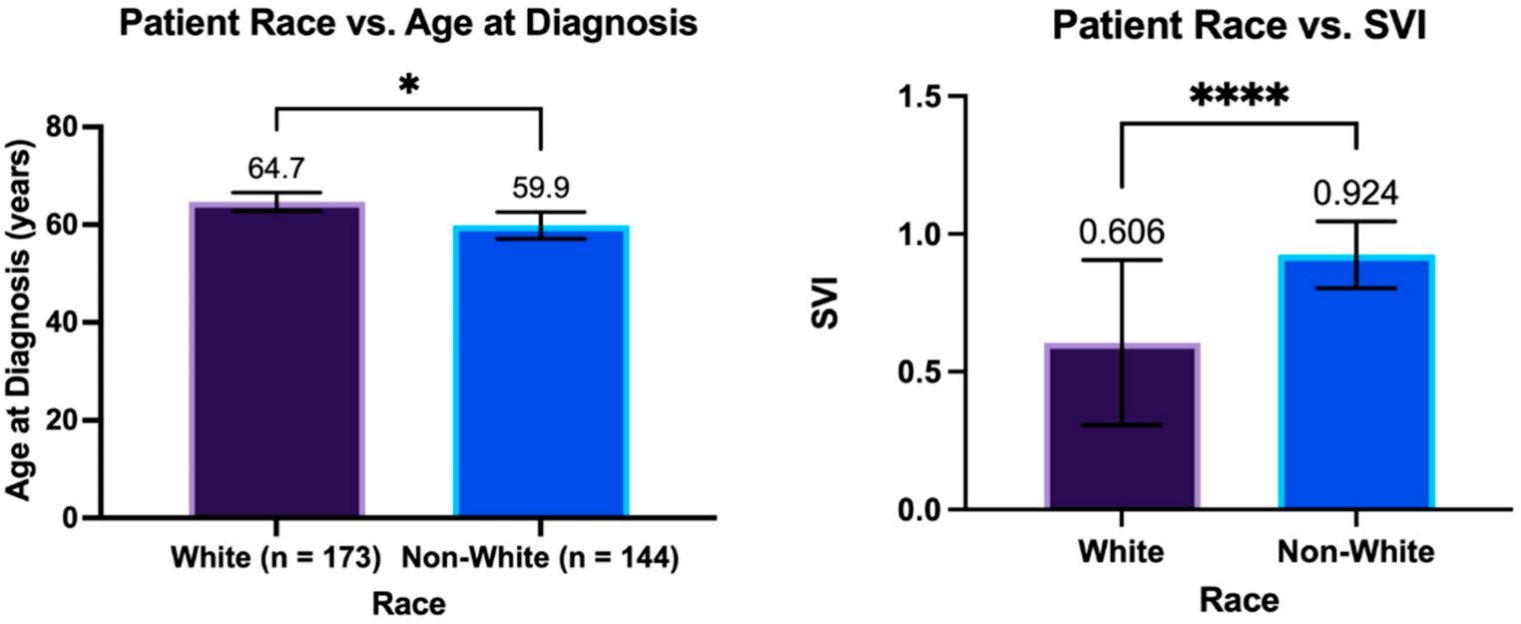
The relationship between White race and (**A**) Age at diagnosis of HGG and (**B**) SVI*; p-value of < 0.05 denotes statistical significance *SVI = Social Vulnerability Index

**Fig. 2:**
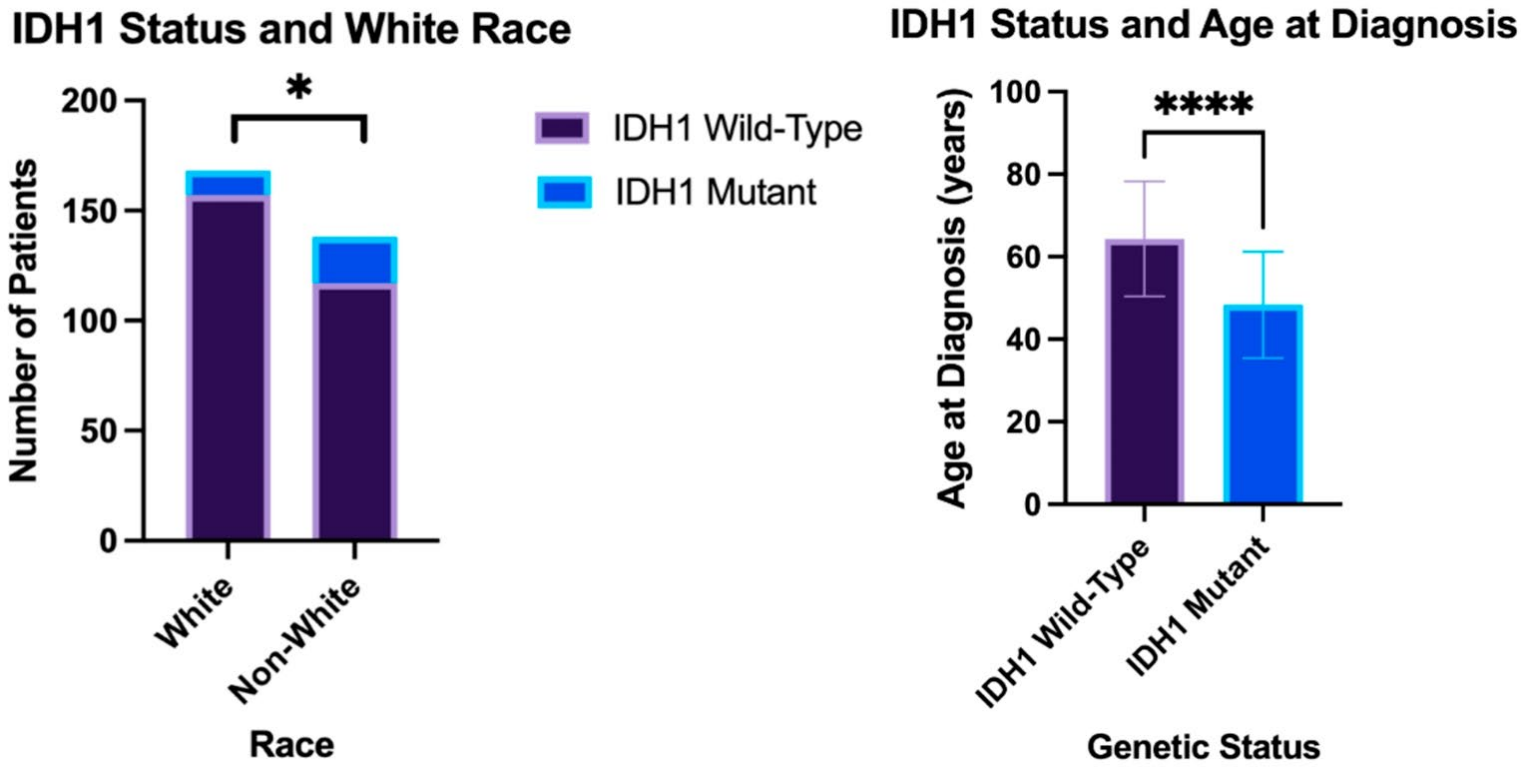
The relationship between IDH1* status and (**A**) White Race and (**B**) Age at diagnosis; p-value of < 0.05 denotes statistical significance *IDH1 = Isocitrate Dehydrogenase 1

White patients were shown to have a significantly lower SVI than non-White patients (Fig. 1A.606 +/- 0.299 [*n* = 14] vs. 0.924 +/- 0.122 [*n* = 58]; *p* < 0.0001***) and a later age at diagnosis of HGG (Figs. 1B and 64.7 +/- 12.6 years [*n* = 173] vs. 59.9 +/- 16.7 years [*n* = 144]; *p* = 0.03*). However, there was no independent association between SVI and age at diagnosis. Independently, White race was associated with an increased likelihood of being IDH1 wild-type (93.5% vs. 84.8% [*n* = 306]; *p* = 0.02*), while IDH1 wild-type patients independently had a later age at diagnosis (64.3 +/- 14.0 years vs. 48.3 +/- 12.9 years [*n* = 307]; *p* < 0.0001****) than IDH1 mutants (Fig. 2). On multivariate analysis using IDH1 status and White race, both factors were still significantly associated with a later age at diagnosis (*p* < 0.0001****; *p* = 0.04*).

### Variations in clinical presentation

**Fig. 3:**
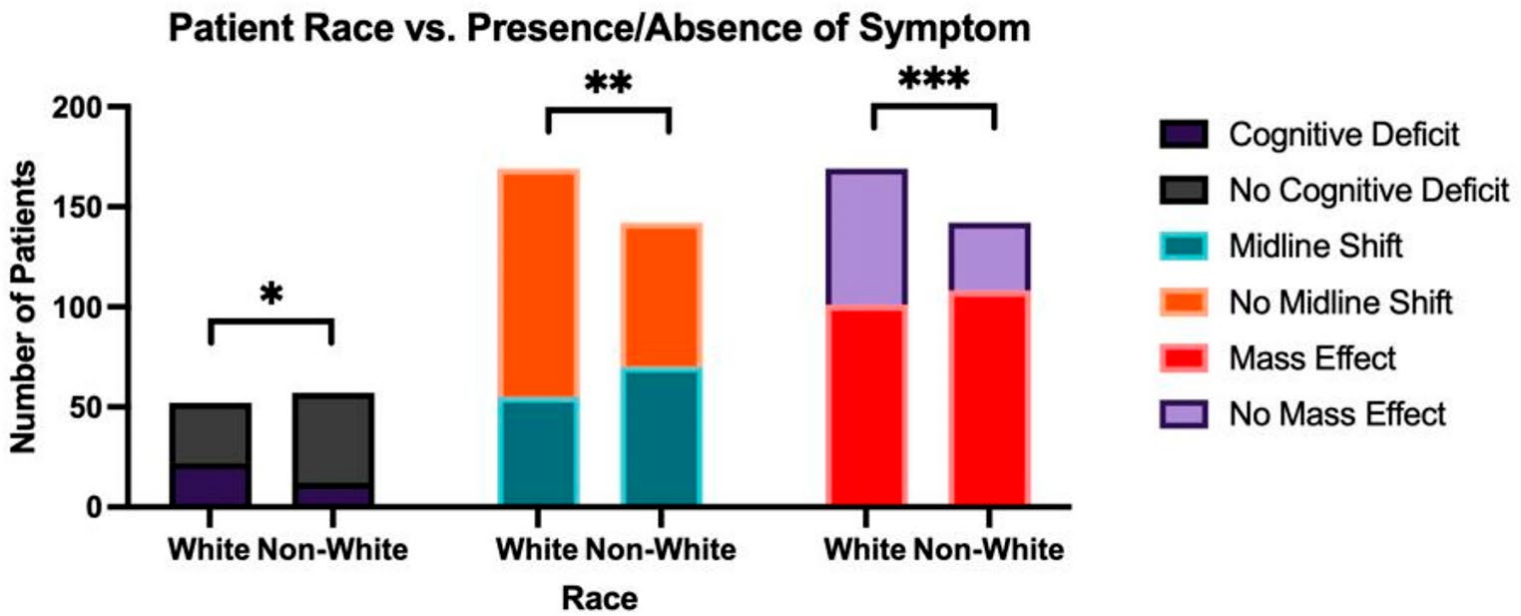
The relationship between White race and presence or absence symptoms at presentation of HGG*, including cognitive deficit, midline shift, and mass effect; p-value of < 0.05 denotes statistical significance *HGG = High-Grade Glioma

**Fig. 4:**
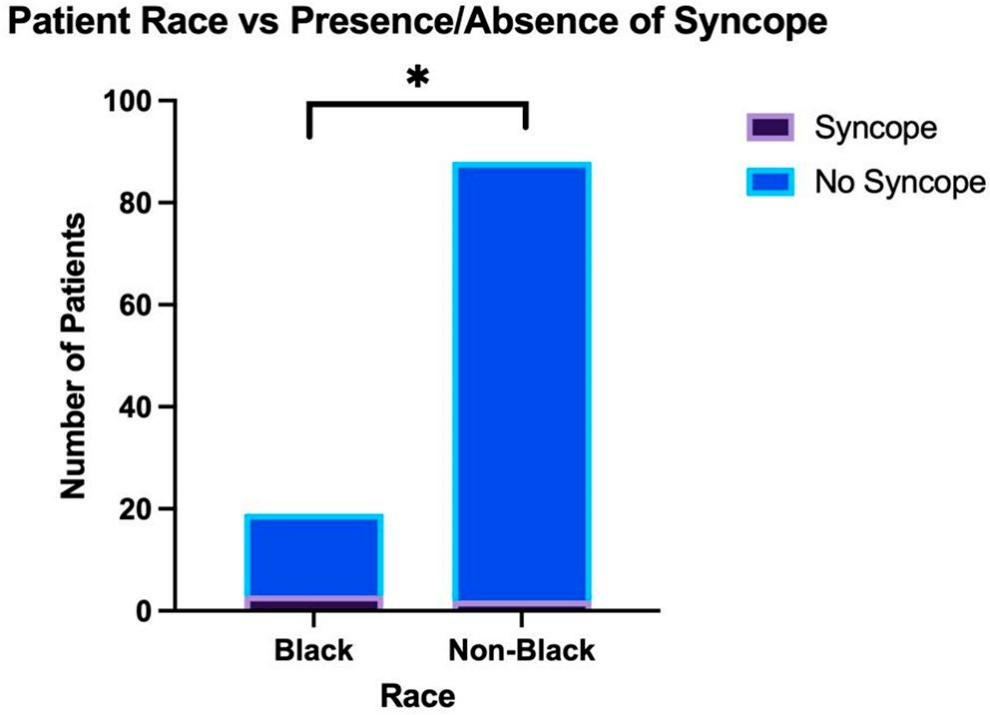
The relationship between Black race and presence or absence of syncope at presentation of HGG; p-value of < 0.05 denotes statistical significance

**Fig. 5:**
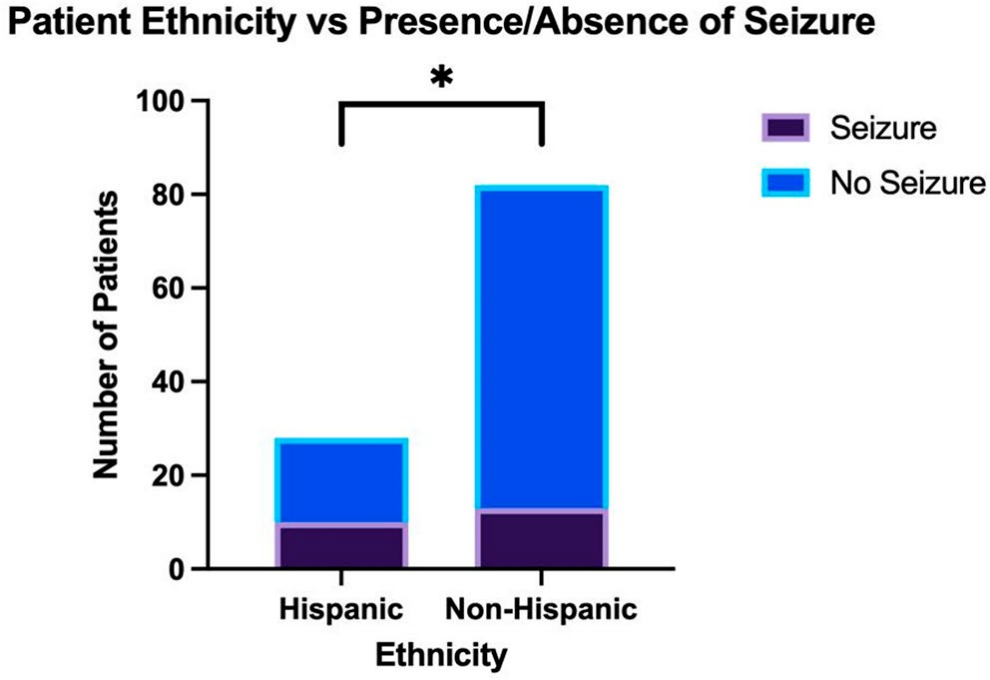
The relationship between Hispanic ethnicity and presence or absence of seizure at presentation of HGG; p-value of < 0.05 denotes statistical significance

Using Fisher’s exact test to analyze differences in initial patient presentation compared to expected values, White patients were more likely to present with a cognitive deficit (Figs. 3 and 42.3% vs. 21.1% [*n* = 109]; *p* = 0.02*) while less likely to present with midline shift (Figs. 3 and 32.5% vs. 49.3% [*n* = 311]; *p* = 0.004**) and mass effect on imaging (Figs. 3 and 59.8% vs. 76.1% [*n* = 311]; *p* = 0.003***) when compared to non-White patients. On the other hand, Black patients were more likely to present with syncope than non-Black patients (Figs. 4 and 15.8% vs. 2.3% [*n* = 107]; *p* = 0.04*). Furthermore, Hispanic patients were more likely to present with seizure than non-Hispanic patients (Figs. 5 and 35.7% vs. 15.9% [*n* = 110]; *p* = 0.03*). Of note, the analysis on Hispanic patients was carried out using the patients who reported a Hispanic ethnicity rather than a Hispanic race, as the former included a larger subset of patients who reported “Other” race in addition to those who reported a Hispanic race. No race-related or ethnicity-related differences were observed in other clinical symptoms, including memory loss, visual disturbances, auditory disturbances, language deficit, altered mental status, motor weakness, and personality changes. Furthermore, no race-related or ethnicity-related differences were observed in KPS score at presentation. Finally, mean SVI was compared between groups with and without a certain presenting sign/symptom, and no significant inter-group difference was found.

## Discussion

Many studies have been conducted that highlight the association between race and glioma incidence, outcomes and survival [20–23]. In the United States, incidence of glioma is greatest among non-Hispanic Whites [13]. Around the world, incidence of glioma is greatest in the USA, Canada, Australia, and Northern Europe [17]. Many studies also exist highlighting differences in outcomes as they relate to sociodemographic background, including one from our own institution finding that recurrence of HGG is delayed in Hispanic patients despite increased social vulnerability [24]. However, few studies exist on the differences in clinical presentation of HGG as they relate sociodemographic background. Data from the patient population at Montefiore Medical Center, which treats a majority of underserved patients and those who are part of racial and ethnic minorities, compared with data from two other institutions treating primarily White patients, provides a unique opportunity to identify the role race and ethnicity plays in the clinical presentation, course, and outcomes of HGG. In this study, in addition to examining social vulnerability and age at diagnosis for HGG, we sought to analyze how sociodemographic background may be associated with differences in clinical presentation of HGG.

Using SVI, we found that White patients were less socially vulnerable than non-White patients. This analysis was conducted using patients from our institution alone and demonstrated that even within a single borough of New York City, there is considerable variation in social vulnerability that may have a racial component. Prior studies have been done on how social factors impact outcomes and survival in brain tumor patients. A study done by Jiminez et al. demonstrated that presence of social determinants of health (SDOH) disparity, which shares considerable overlap with SVI, is associated with an increased hospital length of stay and increased 90-day mortality in patients with brain tumors [25]. However, another study done by Kasl et al. specifically on glioblastoma patients demonstrated no association between socioeconomic status and prognosis in patients with glioblastoma [26]. While that study only examined socioeconomic status, which is one component of SVI, it demonstrates that the impact of social and economic factors on brain tumor outcomes is variable. More studies need to be conducted that analyze the association between social vulnerability and HGG prognosis.

In our analysis, we found that socially less vulnerable White patients were diagnosed with HGG nearly 5 years later than non-White patients, on average. While this trend may be explained by differences in social vulnerability rather than racial differences, in our study, SVI had no association with age at diagnosis, implying a significant role of race in age at diagnosis of HGG. This, for the most part, is consistent with prior studies that showed that White patients generally have a later age at diagnosis than non-White patients [17, 23, 27]. On the flip side, one large, population-based study including over 1,500 patients demonstrated no racial difference in age at diagnosis of GBM [23]. Furthermore, it is well established that patients who are IDH1 wild-type have a later age at diagnosis as well [28, 29], and it is true that in our cohort, white patients were more likely to be IDH1 wild-type. As these two factors are potentially confounding, a multivariate analysis was done to show that White race was associated with a later age at diagnosis, independent of IDH1 status.

As far as how age at diagnosis impacts survival, multiple studies have shown that a younger age at diagnosis correlates with improved survival in glioblastoma and HGG in general [30–33]. There are a few possible explanations for this phenomenon. As expected, younger patients have far less co-morbidities than older patients, giving them a better chance to handle a new cancer. Secondly, younger patients are enrolled in research trials disproportionately more frequently [34, 35] and, in a similar vein, are treated far more aggressively [30].

The current study focuses on sociodemographic differences in clinical presentation of HGG. In our analyses, we found that White patients were more likely to present with a cognitive deficit while less likely to present with midline shift and mass effect on imaging than non-White patients. These findings are bolstered by the fact that there was no significant association between SVI and presence or absence of any of the clinical signs and symptoms examined in this study. This suggests that factors such as lack of healthcare access and agency may not have played a role in the presence of more aggressive symptoms at presentation. Interestingly, the literature is sparse on the variation in clinical presentation in HGG among different racial and ethnic groups. One hypothesis for the results observed in our study is that White patients may develop less aggressive and slower growing tumors. As a result, they present with more indolent symptoms (cognitive deficit) rather than more severe symptoms such as midline shift or mass effect. Furthermore, brain tumor patients with high-risk symptoms, such as focal neurological deficits, may be diagnosed more quickly than patients with lower-risk and nonspecific symptoms [36]. Why this effect was enhanced in White patients in our study is unknown, and it warrants further studies with larger sample sizes.

Our study also found that Black patients were more likely to present with syncope than non-Black patients, and Hispanic patients were more likely to present with seizures than non-Hispanic patients. This marks a difference in presentation from White patients, as Black and Hispanic patients with HGG presented with more severe symptoms. Studies examining syncope in glioblastoma and brain tumors as a whole are primarily limited to case reports. Three case reports highlight patients who had syncope as the sole presenting symptom, and in two of them, the syncope was a result of a seizure [37–39]. Both syncope and seizure appear to be a seemingly rare presenting symptom in the population but occur in a statistically significant subset of our Black and Hispanic patients, respectively.

Another point to note is that even though non-White patients exhibited more radiologically aggressive signs such as midline shift and mass effect, this did not translate into a higher rate of clinical signs and symptoms of increased intracranial pressure, such as severe headache, nausea, vomiting, visual changes, and altered mental status. Therefore, although symptoms that were more prevalent in Black and Hispanic patients, including syncope and seizure respectively, appear to be more severe, perhaps their presence led to earlier presentation to the hospital before further radiographic progression of their tumors occurred. This indicates a potential beneficial impact of alarm symptoms in brain tumors, although more correlation needs to be studied with degree of invasiveness of said tumors.

Factors other than sociodemographic background may impact the clinical presentation of brain tumors. Much data on this is limited to case reports, but these factors include rate of growth, location, and tumor type [40]. As expected, a rapidly-growing tumor, such as HGG, will more likely cause observable symptoms, while a slowly-growing tumor allows time for the brain to adapt, and may be more neurologically silent [41, 42]. One review of 165 brain tumor cases found only marginally significant differences in tumor types between cases that had neurological symptoms concurrent with psychiatric symptoms versus those that had neurologic symptoms only following psychiatric symptoms [40]. When attempting to predict outcomes in such a rare tumor type, it is important to assess a broad range of both modifiable and non-modifiable risk factors.

Limitations of this study include a heterogenous patient population derived from three different institutions, minor inconsistencies in the reporting of race and ethnicity, and reporting of clinical presentation being subject to inter-provider variability in the electronic medical record. Furthermore, IDH status was determined by immunohistochemical staining rather than DNA sequencing, the latter of which can capture non-canonical IDH mutations. Finally, there may have been inconsistencies between the patient’s true SVI at presentation and the SVI data used here, as the SVI data used in this study was based off 2020 census data, while many patients in this study presented before that.

## Conclusion

HGG’s are among the rarest and most devasting neurological malignancies, and recently, there has been more focus on the factors that influence patient outcomes. While there exists literature on the clinical presentation of brain tumors as a whole, there are far fewer studies examining HGG’s specifically, and even less on the role sociodemographic background may play. The goal of this study was to examine the different clinical presentations of HGG as it pertains to sociodemographic background, along with analyzing demographic factors such as age at diagnosis and social vulnerability.

We found that White patients were less socially vulnerable than non-White patients and were diagnosed with HGG at a later age, independent of IDH1 status. Furthermore, they were more likely to present with a milder symptom such as a cognitive deficit, while less likely to present with major symptoms such as mass effect and midline shift on imaging. In addition, Black patients were more likely to present with syncope and Hispanic patients were more likely to present with seizure.

More studies need to be done on how sociodemographic background can impact clinical presentation, and what about those factors can possibly predict presenting symptoms. Furthermore, it would be interesting to see how presenting symptom impacts outcomes in a larger patient cohort, as this could potentially be included in predictive modeling tools.

## Data Availability

No datasets were generated or analysed during the current study.
